# The Influence of the Different Repair Methods on the Electrical Properties of the Normally off p-GaN HEMT

**DOI:** 10.3390/mi12020131

**Published:** 2021-01-26

**Authors:** Di Niu, Quan Wang, Wei Li, Changxi Chen, Jiankai Xu, Lijuan Jiang, Chun Feng, Hongling Xiao, Qian Wang, Xiangang Xu, Xiaoliang Wang

**Affiliations:** 1 Key Lab of Semiconductor Materials Science, Institute of Semiconductors, Chinese Academy of Sciences, Beijing 100083, China; diniu@semi.ac.cn (D.N.); wangquan@semi.ac.cn (Q.W.); wli@semi.ac.cn (W.L.); cxchen@semi.ac.cn (C.C.); jkxu@semi.ac.cn (J.X.); ljjiang@semi.ac.cn (L.J.); cfeng@semi.ac.cn (C.F.); hlxiao@semi.ac.cn (H.X.); qianwang@semi.ac.cn (Q.W.); 2 Center of Materials Science and Optoelectronics Engineering, University of Chinese Academy of Sciences, Beijing 100049, China; 3 School of Microelectronics, University of Chinese Academy of Sciences, Beijing 100049, China; 4 Beijing Key Laboratory of Low Dimensional Semiconductor Materials and Devices, Beijing 100083, China; 5 State Key Laboratory of Crystal Materials, Shandong University, Jinan 250100, China; xxu@sdu.edu.cn

**Keywords:** p-GaN high-electron-mobility transistor (HEMT), different repair methods, repair process

## Abstract

The influence of the repair process on the electrical properties of the normally off p-GaN high-electron-mobility transistor (HEMT) is studied in detail in this paper. We find that the etching process will cause the two-dimensional electron gas (2DEG) and the mobility of the p-GaN HEMT to decrease. However, the repair process will gradually recover the electrical properties. We study different repair methods and different repair conditions, propose the best repair conditions, and further fabricate the p-GaN HEMTs devices. The threshold voltage of the fabricated device is 1.6 V, the maximum gate voltage is 7 V, and the on-resistance is 23 Ω·mm. The device has a good performance, which proves that the repair conditions can be successfully applied to the fabricate of the p-GaN HEMT devices.

## 1. Introduction

GaN high-electron-mobility transistors (HEMTs) are very suitable for power switching devices due to their high two-dimensional electron gas (2DEG) concentration, high breakdown voltage, and high electron mobility [1,2,3,4,5,6]. However, due to the polarization effect, the traditional AlGaN/GaN HEMTs generally are normally on (depletion-mode) [7]. In order to simplify the circuit and improve the safety, we need some methods to realize the normally off (enhancement-mode) GaN HEMT in practical applications [8]. At present, the main methods for realizing the normally off GaN HEMT include recessed gate [9,10,11], p-GaN cap layer [12,13,14], fluorine-plasma ion implantation [15,16], InGaN cap layer [17,18], and so on [19,20,21]. Among these methods, the most commonly used method is the p-GaN cap layer structure because of its high reliability [22]. In the fabrication processes of the p-GaN HEMT device, the important processes include selective etching of over-grown p-GaN layers and the repair process. The p-GaN HEMT etching process requires a high etching selectivity ratio. Both over-etching and under-etching will affect the performance of the device [23]. In order to improve the etching selectivity ratio, some measures had been proposed. Some researchers controlled the etching rate by changing the radio frequency (RF) bias power, inductively coupled plasma (ICP) power or the chamber pressure [24]. Some researchers had achieved self-terminating technology by changing the etching gas, i.e., [23,25]. Among these methods, the most commonly used method is adding O_2_ into the etching gas (Cl_2_/O_2_/N_2_). When the etching gas reaches the AlGaN layer, it will form the (Al,Ga)O_x_ cluster with the AlGaN layer [25]. The bond energy of the (Al,Ga)O_x_ cluster is relatively high and difficult to be etched away, so the etching selection ratio can be improved [25]. However, studies have found that the etching process will cause Cl ions to enter the epitaxial wafer (exist in (Al,Ga)O_x_ or AlGaN layer), which affects the performance of the epitaxial wafer [23,25]. At the same time, the etching will produce damage, further affecting the performance of the epitaxial wafer [24,26]. Therefore, the repair process is required after the etching process. However, as far as we know, there are relatively few studies on the repairing process, and the mechanism of the repair process has not been studied very clearly. Based on previous studies, this paper first studied in detail the influence of different repair methods on the 2DEG concentration (*N_s_*) and the mobility (*μ*) of the AlGaN/GaN HEMT. Afterwards, based on the optimized repair conditions, repair experiments were carried out on the p-GaN HEMT to verify the effectiveness of the conditions. Finally, we fabricated the repaired p-GaN HEMT devices and tested their performance.

## 2. Device Structure

**Figure 1:**
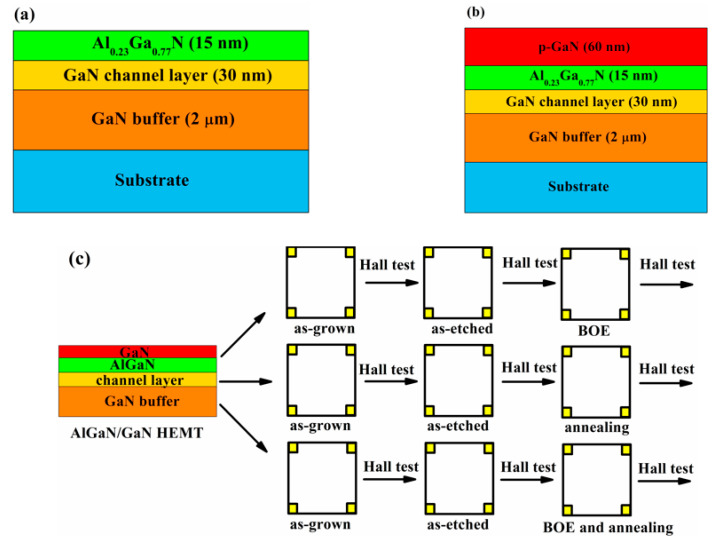
(**a**) Epi-structure of the AlGaN/GaN high-electron-mobility transistor (HEMT), (**b**) epi-structure of the p-GaN high-electron-mobility transistor (HEMT), and (**c**) the experimental steps of the AlGaN/GaN HEMT.

Figure 1a is the epi-structure of the AlGaN/GaN HEMT. The epitaxial structure is grown by the metal–organic chemical vapor deposition (MOCVD) on a 2-inch sapphire substrate. The structure consisted of a 2 μm GaN buffer layer, a 30 nm GaN channel layer, and a 15 nm AlGaN barrier layer (Al composition is 0.23). Figure 1b shows the epi-structure of the p-GaN HEMT with an additional layer of 60 nm p-GaN layer. The doping concentration of the p-GaN layer is about 4 × 10^19^ cm^−3^ and the hole concentration of the p-GaN layer is about 4 × 10^17^ cm^−3^ (annealing at 850 °C in N_2_ ambient for 10 min). The former AlGaN/GaN HEMTs are used to test different repair methods (in order to eliminate the influence of the growing p-GaN layer). Figure 1c shows the experimental steps of the AlGaN/GaN HEMTs. First, make the epitaxial wafer into multiple 10 mm square samples, and then the magnetron sputtering equipment is used to form ohmic contacts at the four corners of the square samples to perform the Hall-effect measurements. The ohmic metal layers are Ti/Al/Ni/Au (20/160/55/50 nm), and then annealing at 870 °C in N_2_ ambient for 30 s. Then, separately perform the Hall-effect measurements on each sample. Afterwards, multiple samples are etched together with the ICP equipment, the etching gas is Cl_2_/N_2_/O_2_. The etching rate of the p-GaN and AlGaN layer is about 10 nm/min, and 1.5 nm/min, respectively. The etching time of the AlGaN/GaN HEMT is 1.5 min, which simulated the case of over-etching the p-GaN HEMT for 1.5 min. After etching, the Hall-effect measurements are separately performed on each sample. After the Hall-effect measurements, different repair methods are used to repair experiments. Then, perform the Hall-effect measurements again after each repair experiment. The p-GaN HEMTs are used to verify the effectiveness of the optimized repair conditions. The steps of the experiment are basically unchanged. The etching time is 7.5 min (over-etching time is 1.5 min). 

## 3. Results and Discussion

**Figure 2:**
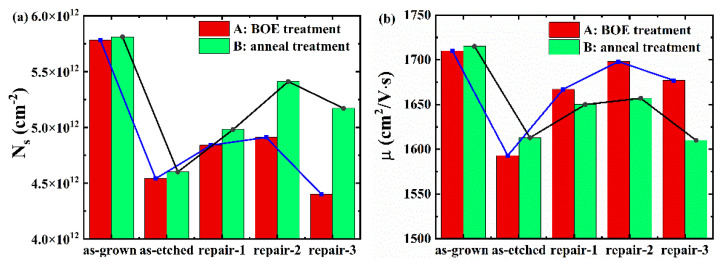
(**a**) The *N_s_* of sample A and sample B in different states, (**b**) the *μ* of sample A and sample B in different states.

**Figure 3:**
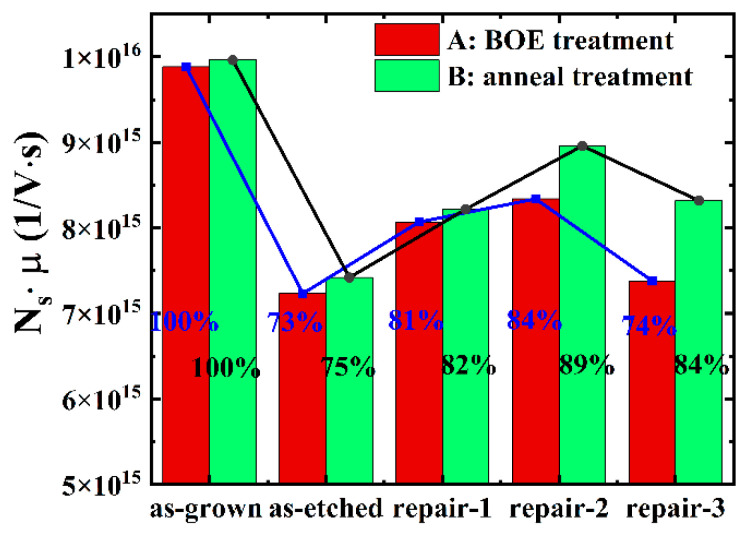
The product of the *N_s_* and the *μ* of sample A and sample B in different states.

Firstly, the influence of the buffered-oxide etchant (BOE) treatment method on the *N_s_* and the *μ* of the AlGaN/GaN HEMT (sample A) is studied. As shown in Figure 1c, first carry out 1 min BOE treatment, and then increase the treatment time by 1 min. The hall-effect measurements are required after each treatment. The red parts in Figure 2a,b show the influence of the etching process and the BOE treatment process on the *N_s_* and the *μ* of sample A. It can be seen that the *N_s_* and the *μ* of sample A after etching (as-etched) are significantly reduced. As the BOE treatment time increases, the *N_s_* and the *μ* of sample A increase (repair-1, 1 min BOE treatment). When the BOE treatment time is 2 min, the *N_s_* and the *μ* of sample A recover to the maximum values (repair-2, 2 min BOE treatment). As the BOE treatment time continues to increase, the *N_s_* and the *μ* begin to decrease again (repair-3, 3 min BOE treatment). The reason may be that the etching will cause Cl ions to enter the epitaxial wafer, which may exist in the AlGaN layer [23] or (Al,Ga)O_x_ layer [25]. Due to the repulsive movement of electrons, the negatively charged Cl ions will affect the *N_s_* of the epitaxial wafer. At the same time, due to the effect of Coulomb scattering, the Cl ions will affect the *μ* of the epitaxial wafer. Therefore, the *N_s_* and the *μ* decrease after etching. After BOE treatment, the (Al,Ga)O_x_ layer of the epitaxial wafer will be removed, and a large amount of Cl ions will be removed, resulting in an increase in the *N_s_* and the *μ*. However, if the BOE treatment time is too long, the hydrofluoric acid (HF) in the BOE will deteriorate the epitaxial wafer, further affecting the performance of the epitaxial wafer. The red parts in Figure 3 show the influence of the etching process and the BOE treatment process on the product of the *N_s_* and the *μ* (the product is related to the device current). It can be seen that the BOE treatment can recover the product value to 84% (repair-2) of the product value before etching (as-grown). 

Secondly, the influence of the annealing method on the *N_s_* and the *μ* of AlGaN/GaN HEMT (sample B) is studied. The experimental steps are shown in Figure 1c. The experimental sample (sample B) and Sample A are small squares of 10 mm at different positions on the same epitaxial wafer. The annealing temperature is 500 °C, the annealing time starts from 1 min, and the annealing time is increased by 2 min. The green parts in Figure 2a,b show the influence of the etching process and the annealing treatment process on the *N_s_* and the *μ* of sample B. It can be seen that the *N_s_* and the *μ* of sample B also decrease to a greater extent after etching (as-etched). As the annealing time increases, the *N_s_* and the *μ* concentration increase (repair-1, 1 min anneal treatment). When the annealing time is 3 min, the *N_s_* and the *μ* of sample B recover to the maximum value (repair-2, 3 min anneal treatment). However, as the annealing time continues to increase, the *N_s_* and the *μ* of the epitaxial wafer begin to decrease (repair-3, 5 min anneal treatment). This may contribute to the following two aspects. On the one hand, annealing can repair the lattice damage (reconstruction of surface stoichiometry) [26], and increase the *N_s_* and the *μ*. On the other hand, at the annealing process, part of the Cl ions on the surface will diffuse into the AlGaN layer (annealing may drive impurity diffusion [26]), further reducing the *N_s_* and the *μ*. Two mechanisms result in a trade-off consideration, and thus an optimal annealing time. The green parts in Figure 3 show the influence of the etching process and the annealing treatment process on the product of the *N_s_* and the *μ*. It can be seen that the annealing treatment can recover the product value to 89% (repair-2) of the product value before etching (as-grown). 

**Figure 4:**
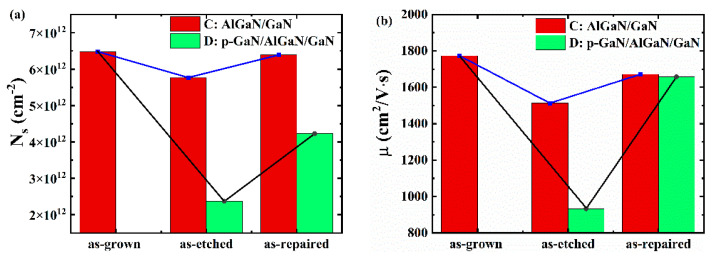
(**a**) The *N_s_* and the *μ* of sample C in different states, (**b**) the *N_s_* and the *μ* of sample D in different states.

**Figure 5:**
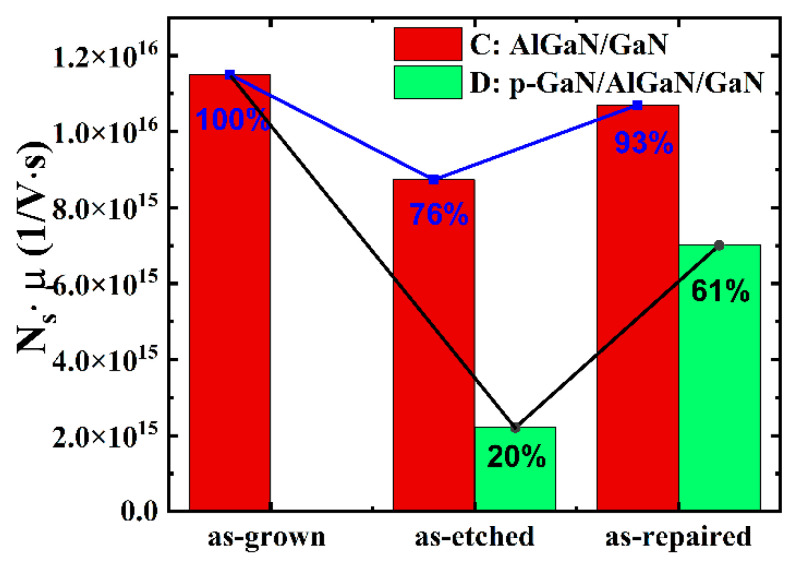
The product of the *N_s_* and the *μ* of sample C and sample D in different states.

Then, combine the two methods mentioned above to repair the damage of the devices. We first conduct the BOE treatment for 2 min, and then annealing at 500 °C for 3 min. The red parts in Figure 4a,b show the *N_s_* and the *μ* of the AlGaN/GaN HEMT (sample C) in different states (as-grown, as-etched, and as-repaired). It is obvious that there is the same trend as the above experiment, and after the two treatments, the electronic properties of the epitaxial wafer have increased to a large extent. The red parts of Figure 5 show the product of the *N_s_* and the *μ* of sample C in different states. The combined method can recover the product value to 93% of the product value before etching (as-grown).

Furthermore, according to the experimental results of the AlGaN/GaN HEMTs (sample A, sample B, and sample C), we conduct a repair study on the p-GaN HEMT (sample D, the structure has been shown in Figure 1b). The etching time is 7.5 min (the over-etching time is 1.5 min, which is the same as the etching time of the AlGaN/GaN HEMTs). Under the same experimental steps and repair conditions (BOE for 2 min, annealing at 500 °C for 3 min), we study the influence of the etching process and the repair process on the *N_s_* and the *μ* of sample D. It can be seen from the green parts of Figure 4a,b that, compared with sample C (as-grown), the *N_s_* of the completely etched sample D (as-etched) is reduced by approximately 70%. In addition, the *μ* is reduced by approximately 47% (compare sample C (as-grown)). After repair (as-repaired), the *N_s_* and the *μ* increased by 64% and 75% (compare sample C (as-grown)), respectively. The green parts of Figure 5 show the product of the *N_s_* and the *μ* of sample D in different states. The product value decreases after etching and recovers to 61% after repairing (compare with sample C (as-grown)). It can be seen that under the same repairing conditions, the recovery degree of sample D is less than that of sample C (the recovery degree reaches 93%). The difference in the recovery degree may be caused by the different thickness of the remaining AlGaN layer after etching. However, the trends shown are consistent.

**Figure 6:**
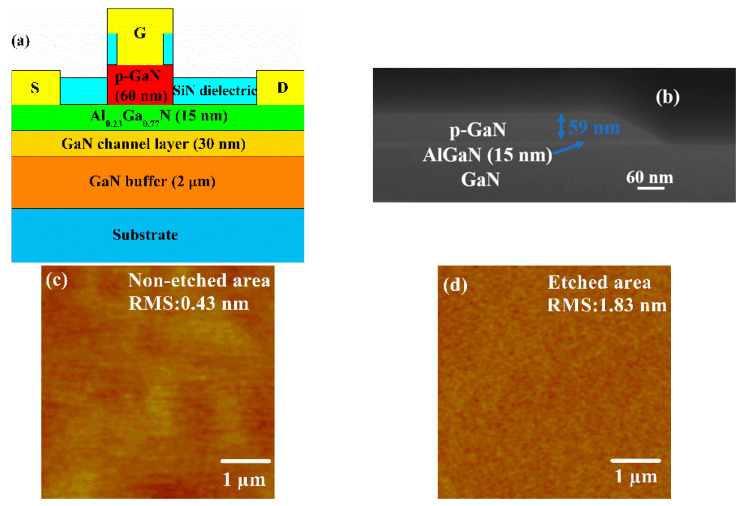
(**a**) Cross-sectional structure of the p-GaN HEMT device, (**b**) cross-section focused ion beam (FIB) image of the p-GaN HEMT after complete p-GaN removal from access regions, (**c**) surface morphology of the p-GaN HEMT non-etched area, (**d**) surface morphology of the p-GaN HEMT etched area.

Finally, we fabricate the p-GaN HEMT device, the schematic cross-sectional structure of the p-GaN HEMT device is shown in Figure 6a. The device fabrication starts with the mesa isolation. Then, the ICP equipment is used to etch the p-GaN layer in non-gate area (p-GaN layer remains the length of 3 μm), and the etching gases are Cl_2_/N_2_/O_2_. The etching time is 7.5 min (over-etching 1.5 min). Figure 6b shows the cross section near the gate region of the p-GaN HEMT in the focused ion beam (FIB) after etching. It can be clearly seen that there is a step of about 60 nm. Figure 6c,d are the surface morphology of the p-GaN HEMT non-etched and etched area in the atomic-force microscope (AFM), respectively. It can be seen that the Root Mean Square (RMS) roughness of the etched area is significantly increased. Then, the BOE treatment is carried out for 2 min, and 500 °C annealing is carried out for 3 min. After that, the metal layers Ti/Al/Ni/Au (20/160/55/50 nm) are deposited by the magnetron sputtering, and then annealing at 870 °C in N_2_ ambient for 30 s in order to form ohmic contacts. Then the plasma-enhanced chemical vapor deposition (PECVD) equipment is used to deposit the SiN_x_ dielectric layer, and the reactive ion etching (RIE) equipment is used to define the source contact, the gate contact (etching length is 2 μm), and the drain contact. The gate metal is Ni/Au. The device has a gate length (*L_g_*) of 3 μm, a gate width (*W_g_*) of 100 μm, a gate-source spacing (*L_gs_*) of 5 μm, and a gate-drain spacing (*L_gd_*) of 10 μm. 

**Figure 7:**
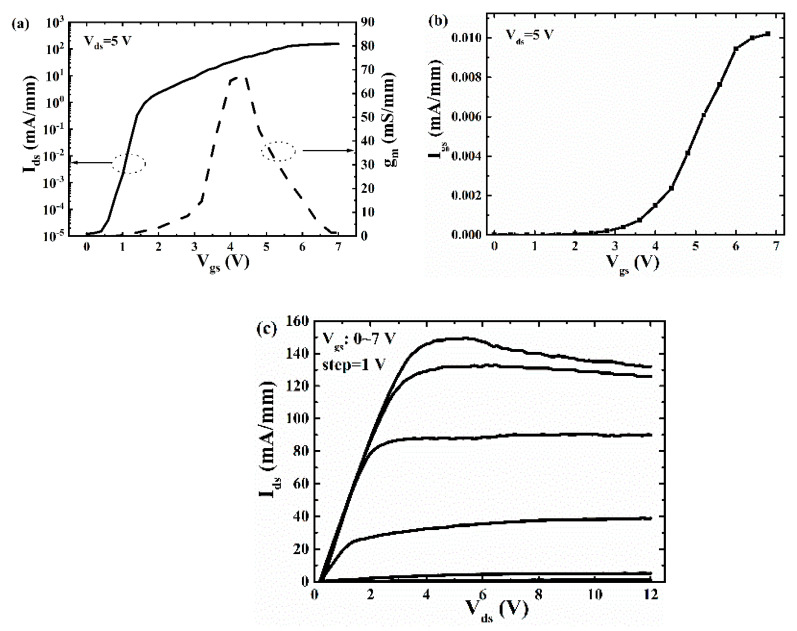
(**a**) Transfer characteristic curves, (**b**) *I_gs_*–*V_gs_* curves, (**c**) output characteristics of the p-GaN HEMT device.

Figure 7 shows the *I*–*V* curves of the repaired p-GaN HEMTs. Figure 7a shows that the threshold voltage (*V_th_*) of the repaired device is 1.6 V (defined as the *I_ds_* = 1 mA/mm [27]), and the max transconductance (*g_max_*) is 68 mS/mm (at *V_gs_* = 4.4 V). The *I_on_*/*I_off_* ratio is about 10^7^. It can be seen from the Figure 7b that when the gate leakage current reaches 0.01 mA/mm, the maximum gate voltage (*V_gs, max_*) is 7 V. The maximum current (*I_d, max_*) is 153 mA/mm (seen from the Figure 7c), the on-resistance (*R_on_*) obtained from the slope of the output characteristics curves is 23 Ω·mm at *V_gs_* = 7 V. At the same time, it can be seen that when *V_gs_* = 7 V, the output current decreases, which may be due to the influence of self-heating effects [28]. Table 1 summarizes and compares the performance of the traditional p-GaN HEMTs fabricated in this paper and other research institutions. It can be seen that the fabricated device has a large *V_th_* and a large *V_gs, max_*. At the same time, it can be seen that the *I_d, max_* of the fabricated device is smaller than that of other research institutions. This is because the *L_g_*, *L_gs_*, and *L_gd_* of the fabricated device are relatively large. 

## 4. Conclusions

In summary, this paper has thoroughly studied the influence of the repair process on the electrical properties of the AlGaN/GaN HEMTs and the p-GaN HEMT. We analyzed the possible mechanisms in different repair methods and optimized the repair conditions. Using the optimized conditions (BOE for 2 min and annealing 500 °C for 3 min), the product of the *N_s_* and the *μ* of the AlGaN/GaN HEMT can be recovered by 93% (compare with sample C (as-grown)), and the product of the *N_s_* and the *μ* of the p-GaN HEMT can be recovered by 61% (compare with sample C (as-grown)). Furthermore, we fabricated the p-GaN HEMTs, and the device (as-repaired) has a good performance. The repair research in this paper is of great significance for p-GaN HEMT device fabrication.

## Figures and Tables

**Table 1:** Summary and comparison of the performance of the traditional p-GaN high-electron-mobility transistors (HEMTs).

Main Research Institute	Structural Parameters (μm)	*V_th_* (V)	*V_gs, max_* (V)	*R_on_* (Ω·mm)	*I_d, max_* (mA/mm)
Our work	*L*_g_ = 3, *L*_gs_ = 5, *L*_gd_ = 10	1.6	7	23	153
National Tsing Hua University [14]	*L*_g_ = 1, *L*_gs_ = 1, *L*_gd_ = 3	0.5	5	8.2	215.9
Chang-Gung University [27]	*L*_g_ = 3, *L*_gs_ = 2, *L*_gd_ = 7	2.1	8	5.65	272
Samsung [29]	*L*_g_ = 4, *L*_gs_ = 2, *L*_gd_ = 12	1.23	7	14	230
Samsung [30]	*L*_g_ = 4, *L*_gs_ = 2, *L*_gd_ = 9	0.93	8	16	309
